# Dexamethasone versus 5-HT3 receptor antagonists in preventing nausea during awake craniotomy: a propensity score matching study

**DOI:** 10.1186/s40981-024-00746-9

**Published:** 2024-10-07

**Authors:** Takehito Sato

**Affiliations:** Department of Anesthesiology, Nagoya University Hospital, Nagoya City, Aichi, 466-8550 Japan

**Keywords:** Nausea, Vomiting, Awake craniotomy, Propofol, Granisetron, Dexamethasone

## Abstract

**Background:**

Nausea and vomiting during awake craniotomy (AC) can increase cerebral pressure and cause asphyxia and aspiration. 5-HT3 receptor antagonists, such as granisetron, are often administered before awakening to prevent nausea during AC. Recently, dexamethasone was reported to prevent nausea and vomiting during AC; however, the efficacy of both drugs in preventing nausea has not yet been investigated.

**Methods:**

We examined the frequency of nausea and vomiting in AC patients (*n* = 170) treated at our hospital until the end of September 2019. We divided patients as those who received dexamethasone (*n* = 71) and or granisetron (*n* = 99) before awakening and examined the frequency of nausea and vomiting after propensity score (PS) matching.

**Result:**

Eighty-two patients were selected after PS matching. The incidence of nausea was significantly lower in the dexamethasone group than in the granisetron group (9.8% vs 41.5%, *p* = 0.002). In the logistic regression analysis after matching, the incidence of nausea significantly reduced with dexamethasone treatment (odds ratio: 0.12, 95% confidence interval: 0.029–0.499, *p* = 0.03).

**Conclusion:**

In conclusion, dexamethasone was more effective than granisetron in preventing nausea during AC.

## Introduction

Awake craniotomy (AC) is often performed in patients with brain tumors in regions linked to language processing to minimize any damage to language functioning [1]. AC is typically performed under general anesthesia, with patients waking during surgery for a language task (awake phase) in Japan [2]. Nausea and vomiting during AC are concerning because they can cause increased cerebral pressure, asphyxia, and aspiration. 5-HT3 receptor antagonists, such as granisetron, are often administered before awakening to prevent nausea during AC [3]. Recently, dexamethasone was reported to prevent nausea during AC [4]; however, the superiority of both drugs in preventing nausea has not been investigated.

In this study, we compared efficacies of dexamethasone and granisetron in preventing nausea and vomiting in two groups of patients who underwent AC. We hypothesized that dexamethasone causes fewer nausea and vomiting events than does blocking after general anesthesia during AC.

## Methods

This study was approved by the Ethics Committee of Nagoya University (approval number: 2019–0324). Informed consent was obtained from all participants using the opt-out method. Patients could refuse or opt out of data being held in the study.

Inclusion criteria were as follows: (1) brain tumor in the frontal or temporal lobe requiring intraoperative language tasks or focal resection for epilepsy near the language areas, therefore needing to be awake during the resection, and (2) aged between 18 and 70 years. Exclusion criteria were as follows: (1) American Society of Anesthesiologists (ASA) physical status score above 3, (2) liver failure with Child–Pugh classification B or C, (3) severe respiratory disease (such as severe obstructive pulmonary disease) and respiratory dysfunction (vital capacity < 60% or forced expiratory volume in 1 s < 50%), (4) hemostatic dysfunction (platelet count < 80,000/μL, PT-INR > 1.5, or APTT > 40 s), (5) metoclopramide was administered prophylactically before awake phase, and (6) both granisetron and dexamethasone were administered.

We retrospectively investigated 175 cases of AC performed at Nagoya University Hospital between January 1, 2006, and June 30, 2018. Five patients were excluded: two owing to undergoing incomplete AC because of airway complications (supraglottic device; SGA did not fit) and three owing to severe agitation during the awake phase. We divided the patients into two groups, either receiving granisetron (Group G, *n* = 99) or dexamethasone (Group D, *n* = 71) before the awake phase.

General anesthesia was maintained with target-controlled infusion of propofol 2.5 − 4.6 µg/mL, remifentanil 0.05 − 0.25 µg/kg/min, and fentanyl and rocuronium 10 − 40 mg for maintaining bispectral index between 40 and 60, after insertion of the SGA device (i-gel® or laryngeal mask airway Proseal®). All patients underwent general anesthesia and scalp blocks: 0.375% ropivacaine was used for scalp blocks and the headpin site, surgical site, temporal muscle, and dura. Bilateral scalp blocks (supraorbital nerve, supratrochlear nerve, greater occipital nerve, lesser occipital nerve, auriculotemporal nerve, and zygomaticotemporal nerve) were performed to minimize intraoperative pain before the operation [2, 5, 6]. Analgesia was adjusted to maintain the systolic blood pressure < 140 mmHg during the sleep phase of the procedure. Oxygenation was maintained such that it did not fall below 94%, and the heart rate was 50–100 beats per minute.

Before the awake phase, we administered dexamethasone 6.6 mg or granisetron 3 mg at the discretion of the anesthesiologists, in addition to mannitol 150–300 mL. Once the neurosurgeon could visualize the brain lesion, we stopped infusion of anesthetics and removed the SGA after the patients woke up and could respond to verbal communication. Metoclopramide 10 mg was administered for nausea. If the patients complained of pain with Numerical Rating Scale score > 4, 1% lidocaine with adrenaline was subcutaneously injected: flurbiprofen 50 mg, acetaminophen 1000 mg or 15 mg/kg (if body weight < 50 kg), or fentanyl 25 μg were administered. Intraoperative hypertension was defined as elevated blood pressure > 20% of the baseline value before anesthesia or systolic blood pressure > 150 mmHg during the awake phase. After tumor resection, patients underwent intraoperative magnetic resonance imaging (MRI), followed by induction of general anesthesia. The SGA was inserted again via a lateral caudal approach. At the end of the operation, the patient was awakened from the anesthesia and transferred to the intensive care unit.

The primary outcome was whether intraoperative dexamethasone administration during awake tumor resection reduced the frequency of nausea and vomiting compared with 5-HT3 receptor antagonists.

Propensity score matching was used to control for factors that may influence intraoperative nausea and vomiting during AC. The two groups were matched in a 1:1 ratio (*n* = 41 for each group) through propensity score matching analysis adjusted for six covariates (age, sex, BMI, awake time, dose of fentanyl before awake phase, dose of ropivacaine, highest value of sABP). The nearest-neighbor matching method (1:1 ratio) was applied with a caliper width of 0.2 for the logit-transformed propensity score. Variables in a matched dataset were considered balanced between groups if the standardized mean difference was < 0.1. This model yielded a c-statistic of 0.82, indicating the ability to differentiate between Groups D and G. Categorical variables are expressed as number (percentage) and continuous variables as median (interquartile range). Data were analyzed using Fisher’s exact test and the Mann–Whitney *U*-test. Logistic regression analysis was performed to evaluate factors influencing intraoperative nausea.

Differences were considered significant at *p* < 0.05. Statistical analyses were performed with the statistical analysis software EZR [7] and R version 2.5–1 (The R Foundation for Statistical Computing, Vienna, Austria).

## Results

Before PS matching, the overall incidence of nausea and vomiting were 24.7% and 4.7%, respectively. The incidence of nausea was significantly lower in the dexamethasone group than in the granisetron group (14.1% vs 32.3%, *p* = 0.007). However, significant differences were observed between the two groups in patient demographic factors that may be associated with nausea during awake phase, including fentanyl dose, pain, intraoperative awake time, and ropivacaine dose.

After PS matching, 82 patients were allocated to Group D (*n* = 41) or Group G (*n* = 41). There were no significant differences in patient background factors other than anesthesia time; the incidence of nausea was significantly lower in the dexamethasone group than in the granisetron group (9.8% vs 41.5%, *p* = 0.002) after PS matching. In the logistic regression analysis after matching, the incidence of nausea significantly reduced with dexamethasone treatment (odds ratio: 0.12, 95% confidence interval: 0.029–0.499, *p* = 0.03). However, no significant differences were observed in vomiting incidence before and after matching (Table 1).

**Table 1:** Baseline characteristics and intraoperative data of the two groups before and after propensity score matching

	Before propensity matching			After propensity matching		
	Group G	Group D	*P* value	Group G	Group D	*P* value
Age (Years)	42.0 [36.0, 50.0]	42.0 [33.0, 53.5]	0.58	40.00 [37.0,48.0]	44.00 [35.0,54.0]	0.48
Sex (man/female	34/65	29/42	0.42	15/26	15/26	1.0
Height (cm)	166.30 [161.0,171.4]	167.00 [159.0,171.0]	0.67	165.00 [157.9,170.0]	168.0 [160.0, 172.0]	0.64
Weight(kg)	64.00 [55.0, 72.5]	61.60 [54.3, 67.4]	0.71	58.90 [51.00, 69.00]	61.70 [54.9, 66.3]	0.71
BMI(kg/m^2^)	22.50 [20.1, 25.5]	21.79 [20.2,23.5]	0.12	21.80 [19.0, 23.6]	21.40 [20.3, 23.4]	0.94
Operation time (min)	453.00 [395.5,550.5]	435.00 [377.5,502.5]	0.086	425.0 [388.0, 492.0]	410.0 [367.0, 490.0]	0.28
Awake time (min)	163.0 [124.0,226.5]	134.5 [105.5,171.5]	0.004	146.00 [110.0, 173.0]	137.0 [113.0, 164.0]	0.64
Anesthesia time (min)	598.0 [535.0,688.0]	539.0 [480.5,606.5]	<0.001	573.00 [521.0, 65300]	519.0 [472.0, 600.0]	0.016
Bleeding (ml)	454.00 [315.5,762.0]	311.00 [173.5, 580.5]	0.003	432.0 [318.0, 888.00]	272.0 [172.0, 530.0]	0.007
Infusion (ml)	3600.0 [2876.5,4300.0]	3500.00 [3025.0,4015.0]	0.57	3653.0 [3150.0,4250.0]	3350.0 [2950.0, 3800.0]	0.13
Fentanyl before awake phase (μg)	200.0 [0.00, 287.50]	200.0 [200.0, 250.0]	0.028	200.0 [100.0, 300.0]	200.0 [150.0, 200.0]	0.32
Dose of Ropivacaine(mg/kg)	3.00 [2.10, 3.95]	4.30 [3.79, 4.96]	<0.001	3.84 [3.04, 4.98]	4.20 [3.69,4.67]	0.27
Highest Sabp (mmHg)	158.00 [140.5,170.0]	143.0 [132.0,152.5]	<0.001	143.00 [135.0, 160.0]	146.0 [136.0,155.0]	0.68
HT (%)	30(30.3)	39(54.9)	0.002	19(46.3)	18(43.9)	1.00
Nausea (%)	32(32.3)	10 (14.1)	0.007	17(41.5)	4(9.8)	0.002
Vomiting (%)	6(6.1)	2 (2.8)	0.47	4(9.8)	2(4.9)	0.67
Pain (%)	42 (42.4)	20 (28.2)	0.075	16(39.0)	14(34.1)	0.81

Values are presented as medians (interquartile range [IQR])

*p* < 0.05 were considered statistically significant

## Discussion

During awake craniotomy, patients occasionally experience nausea and vomiting because of various factors, such as anesthetic agent effect, psychology (intraoperative anxiety and fear), and inadequate analgesia of the surgical site, dural attachment, and meningeal vessels at awake phase [4].

Reportedly, nausea occurs in 8.3% [8] and 19.3–22% [2, 4] of patients during awake craniotomy. Routine prophylactic preoperative intravenous antiemetics such as dexamethasone and 5-HT3 receptor antagonists can reduce the incidence of this complication. Granisetron is a selective and potent 5-HT3 receptor antagonist for preventing and treating postoperative nausea and vomiting and chemotherapy-induced vomiting in the treatment of malignant diseases [9]. To prevent intraoperative nausea and vomiting, 5-HT3 receptor antagonists are often introduced in AC before the awake phase [3, 10].

No study has compared the efficacy of a 5-HT3 receptor antagonist with that of dexamethasone during the intraoperative management of AC. Other reports indicated that low doses of dexamethasone can prevent intraoperative vomiting. Dexamethasone, a corticosteroid, is a standard agent for treating cerebral edema caused by intracranial tumors. It is widely used in neurosurgical cases as a potent antiemetic with few adverse effects [2, 4, 11]. The mechanism of action to reduce nausea and vomiting is thought to correlate with a decrease in the levels or metabolism of 5-hydroxytryptophan in the central nervous system or a decrease in prostaglandin synthesis [12].

Dexamethasone also has the effect of reducing intracranial pressure [11]; on the other hand, past reports indicate that 5HT3 antagonists do not affect intracranial pressure [13]. And dexamethasone may reduce the inflammatory response by inhibiting the production of inflammatory mediators and reduce the severity of nausea by acting on the neurotransmitter 5-HT and receptor proteins neurokinin (NK) 1 and NK2 [12].

The mechanism of dexamethasone's antiemetic for AC is unclear, but we hypothesized that these mechanisms of dexamethasone appeared to reduce nausea events during AC, although previously reported meta-analyses have reported that, except in neurosurgery, there may be no significant difference between 5HT3 antagonists and dexamethasone in preventing postoperative nausea and vomiting [14]. But this study suggests that owing to these diverse effects, dexamethasone appears to have a stronger antiemetic effect than granisetron, and we found that dexamethasone reduced the incidence of nausea during AC compared with granisetron.

This study had some limitations. First, we did not introduce both dexamethasone and granisetron during the AC. In general, better efficacy could have been achieved by combining antiemetics with different mechanisms of action [4, 5]. Second, this was a retrospective study. Although propensity score matching was performed, future prospective studies are needed. Another limitation is that the effect of intraoperative bleeding cannot be ignored. In this study, blood loss in patients receiving granisetron was approximately 50% larger compared with those receiving dexamethasone, despite similar volume of fluid infusion. Hypovolemia is a risk factor for postoperative nausea and vomiting [15], so which may be one of the reasons for the study results.

In conclusion, intraoperative administration of dexamethasone is more effective than that of granisetron in preventing intraoperative nausea and vomiting during AC.

## Data Availability

They are available as the Excel® file upon reasonable request.

## Abbreviations

AC: Awake craniotomy
BIS index: Bispectral index
NRS: Numeric Rating Scale
sABP: Systolic arterial blood pressure
NK: Neurokinin
